# Associations of body fat percentage-based obesity with depression and comorbidity-related gut microbiota features in college students

**DOI:** 10.3389/fnut.2026.1906822

**Published:** 2026-09-17

**Authors:** Yingli Xu, Zhenchi Niu, Long Zhou, Wenqing Ding, Jing Li

**Affiliations:** School of Public Health, Ningxia Medical University, Yinchuan, China

**Keywords:** body fat percentage, college students, depression, gut microbiota, obesity

## Abstract

**Introduction:**

The association between body fat percentage (BFP)-defined obesity and depression in college students remains unclear, and gut microbiota alterations in this comorbid condition have not been fully characterized.

**Methods:**

A total of 906 college students were recruited to examine the prevalence of comorbid obesity and depression and the association between body fat percentage-defined obesity and depression. Logistic regression was used for association analysis. Subsequently, 104 participants balanced for age and sex distributions across groups were selected and divided into four groups: obese with depression (G1), non-obese with depression (G2), obese without depression (G3), and non-obese without depression (G4). Fecal samples were analyzed by 16S rRNA gene sequencing.

**Results:**

The prevalence of comorbid obesity and depression was 9.71%. BFP-defined obesity was significantly associated with depression after adjustment for covariates (OR = 1.62, 95% CI: 1.10–2.38). Gut microbiota analysis showed no significant differences in α-diversity among groups, whereas β-diversity differed significantly between the G1 and G4 (*P* < 0.05). Descriptive taxonomic profiles suggested differences in the relative abundances of several genera (e.g., decreased *Akkermansia*, increased *Fusobacterium* and *Collinsella* in G1). PICRUSt- based functional prediction suggested differences in inferred microbial metabolic pathways between G1 and other groups, including higher predicted lipopolysaccharide biosynthesis pathway abundance than in G2 and G4.

**Discussion:**

BFP-defined obesity was associated with depression in college students. The microbiome findings were exploratory and suggest that co-occurring obesity and depression may be associated with differences in gut microbial composition and predicted functional potential. Larger longitudinal studies with direct functional and inflammatory measurements are needed.

## 1 Introduction

Depression and obesity are two major public health challenges worldwide, and both have become increasingly prevalent among adolescents, especially college students (1). According to a systematic review and meta-analysis by Lin et al., the prevalence of depression among Chinese university students was 34.7%, and moderate-to-severe depression is associated with an increased risk of suicidal ideation (2). Meanwhile, obesity has risen substantially among emerging adults aged 18–25 years in recent decades (3). A study based on five cycles of the Chinese National Survey on Students' Constitution and Health, including 241,710 college students, reported that the combined prevalence of overweight and obesity increased from 3.7 to 14.0% (4). Depression and obesity frequently co-occur and show a bidirectional association. Systematic reviews indicate that depression is associated with an approximately 50% increased risk of incident obesity, whereas obesity is also linked to a higher risk of subsequent depressive symptoms (5). Their coexistence may result in poorer quality of life and greater physical and psychological burden than either condition alone. College students may be vulnerable to this comorbidity because of academic stress, irregular lifestyles, financial strain, and career uncertainty (6). Body mass index (BMI) is the most widely used anthropometric indicator for defining obesity (7). However, BMI does not distinguish fat mass from lean mass and may therefore misclassify adiposity. The Lancet Diabetes & Endocrinology Commission proposed a more comprehensive diagnostic framework emphasizing the direct effects of excess adiposity on organs and tissues. Body fat percentage (BFP) has been highlighted as one of the more accurate indicators to assess elevated adiposity, particularly in clinical settings (8). However, the prevalence of depression-obesity comorbidity defined by BFP remains unclear in college students.

Beyond their epidemiological coexistence, accumulating evidence suggests that depression and obesity may share common biological mechanisms. The gut microbiome, a complex community of trillions of microorganisms, has emerged as a potential integrative link in the pathophysiology of both conditions (9). In obesity, gut microbiota dysbiosis and impaired intestinal barrier function have been consistently implicated (10). Individuals with obesity have been reported to harbor bacterial taxa with greater pro-inflammatory potential than lean controls (11). Some studies have reported reduced microbial diversity and altered *Firmicutes/Bacteroidetes* ratios in obesity (12, 13). Similarly, depression has been associated with gut dysbiosis, including reduced microbial diversity and altered abundances of specific taxa in patients with major depressive disorder (14, 15). Differentially abundant taxa are commonly reported within the phyla *Bacteroidetes, Firmicutes, Actinobacteria*, and *Proteobacteria* (16) (15, 17). These findings support a key role of the gut microbiota in both obesity and depression and suggest that the two disorders may share microbiota-related mechanisms, including chronic inflammation, hypothalamic-pituitary-adrenal axis dysregulation, and metabolic disturbances (1, 18–20). A previous study identified eight taxonomic biomarkers, mainly within *Firmicutes*, in Mexican adults with co-occurring obesity and depressive symptoms (21). However, the gut microbiota characteristics of college students with comorbid obesity and depression remain poorly understood.

Therefore, this study aimed to examine the association between body fat percentage-defined obesity and depression among college students and to characterize gut microbiota alterations associated with their comorbidity.

## 2 Materials and methods

### 2.1 Study population

This study employed a cross-sectional design and was conducted in Yinchuan, China, in September 2023. Stratified random cluster sampling was used to select three colleges as samples. After stratification by grade and majors, 39 classes were randomly selected. The exclusion criteria were as follows: age < 18 years; current use of any psychotropic medications including antidepressants, anxiolytics, and antipsychotics; and missing data on the Patient Health Questionnaire-9 (PHQ-9), body fat percentage, or covariates. Ultimately, 906 subjects were included. Subsequently, a subset of 104 participants was selected from the initial cohort to achieve approximately comparable age and sex distributions across the four phenotype groups, and fecal samples were collected: (1) the obese with depression group (G1, *n* = 24); (2) the non-obese with depression group (G2, *n* = 27); (3) the obese without depression group (G3, *n* = 27); (4) the non-obese without depression group (G4, *n* = 26). Exclusion criteria were: 1) antibiotic use within 3 months prior to the study; 2) use of laxatives or probiotics within 72 h before fecal sample collection; and 3) presence of diarrhea at the time of sampling. The specific process is shown in Figure 1.

**Figure 1:**
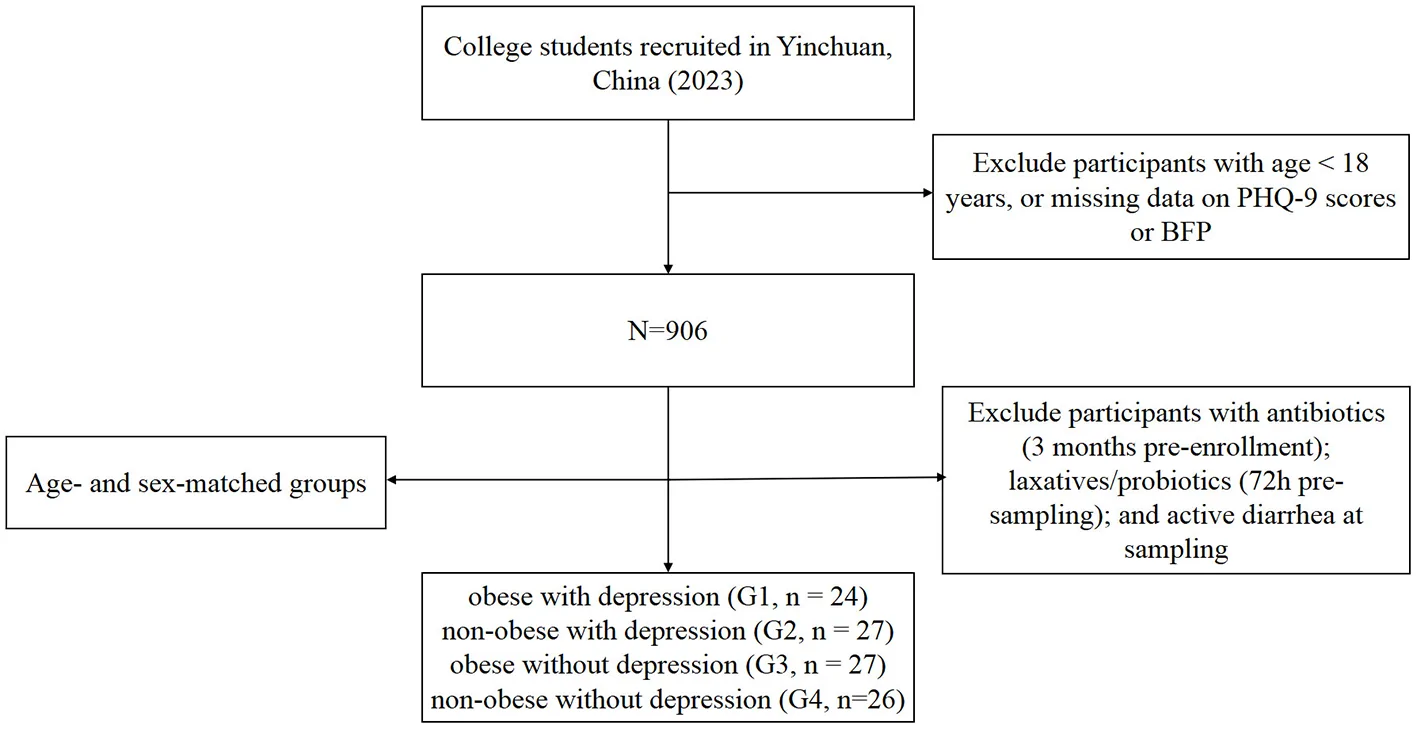
Flow chart.

The study was conducted in accordance with the Declaration of Helsinki and approved by the Ethics Committee of Ningxia Medical University (Approval ID: 2023–009; Approval date: 10 May 2023). Informed consent was obtained from all subjects involved in the study.

### 2.2 The definitions of obesity and depression

All anthropometric measurements were carried out by trained staff using standard protocols. Body composition evaluation through bioelectrical impedance analysis (BIA) was conducted employing the InBody 370 system (Biospace Co., Seoul, Korea), with body fat percentage (BFP) values being directly obtained. According to Satu et al., obesity is defined as a BFP of ≥25% for male and ≥30% for female (22).

Depression was assessed using the 9-item Patient Health Questionnaire (PHQ-9), a validated screening tool. It includes nine items that assess the frequency of depressive symptoms over the previous 2 weeks, with each item scored on a scale of 0 (not at all) to 3 (almost daily). A total score ≥ 10 indicated depression, a cutoff demonstrated to yield high sensitivity and specificity for depression diagnosis (23).

### 2.3 . Stool sample collection and DNA extraction

Before sampling, subjects were provided with dedicated fecal collection tubes and disposable gloves, along with instructions on the sampling procedure. Urinate before defecation to avoid urine contamination. After defecating into a clean container, a sterile spatula was used to collect 3–5 g of feces from the central portion. The sample was then immediately sealed, labeled with the subject's ID and name, and delivered to the collection point within 30 min. The fecal samples collected were frozen immediately in liquid nitrogen and stored at −80 °C until analysis.

Genomic DNA was isolated from samples using the Magbead Soil and Stool DNA Kit (Cwbio, Taizhou, China), according to the manufacturer's instructions. DNA concentration and purity were assessed spectrophotometrically using a Nanodrop, while integrity was evaluated via standard 0.8% agarose gel electrophoresis.

### 2.4 16S rRNA sequencing

Bacterial genomic DNA served as the template for amplifying the hypervariable V3-V4 region of the 16S rRNA gene. Amplification was carried out using the forward primer 5′ -CCTACGGGNGGCWGCAG-3′ and the reverse primer 5′ -GACTACHVGGGTATCTAATCC-3′. Each specimen was subjected to three separate PCR amplifications. Subsequently, the amplification products were analyzed by agarose gel electrophoresis, and products derived from the same sample were combined. The combined amplicons served as the template for index PCR, which utilized indexing primers to incorporate Illumina barcodes into the library. The resulting products were evaluated via gel electrophoresis and purified using DNA Purification Magbeads (Cwbio, Taizhou, China). The purified amplicons constituted the indexed 16S V3-V4 library. Library quality was evaluated using the Qubit^®^ 2.0 Fluorometer (Thermo Scientific) and Agilent Bioanalyzer 2,100 systems. Finally, the pooled library was sequenced on a DNB G99 platform to generate 2 × 300 bp paired-end reads.

### 2.5 Bioinformatics analysis

Gut microbial α-diversity was evaluated using QIIME, computing ACE, Chao1, Observed OTUs (Sobs), and Simpson indices to assess community richness and evenness. Microbial β-diversity was assessed using principal coordinates analysis (PCoA) and analysis of similarities (ANOSIM). PCoA was performed to assess whether significant differences existed in the microbial community composition between the obese-with-depression group and the control groups. Bar charts of taxonomic composition at the phylum and genus levels were used to compare species distribution across groups. LEfSe was used as an exploratory screening approach, using a nominal *P* < 0.05 and an LDA score >2.0. To assess robustness to compositionality and multiple testing, ANCOM-BC2 was additionally applied to each pairwise comparison (G1 vs. G2, G1 vs. G3, and G1 vs. G4). Within each comparison, *P* values were adjusted using the Benjamini-Hochberg procedure, and *q* < 0.05 was considered FDR-controlled statistical significance. PICRUSt was used to infer the relative abundance of KEGG functional pathways from 16S rRNA profiles. These predicted pathways were treated as exploratory estimates of functional potential and not as direct metagenomic, metabolomic, or biochemical measurements.

### 2.6 Covariates

Potential confounders for depression were analyzed, including age, gender, learning burden, drinking, smoking, sleep quality, and physical activity, all assessed with standardized questionnaires. Learning burden was measured by a single self-report question (“Do you perceive your current academic workload as a burden?”) with yes/no responses, coded as heavy (yes) or light (no). Smoking was defined as having smoked at least one cigarette on at least one day during the past 30 days; drinking was defined as having consumed at least one alcoholic drink on at least one day during the past 30 days. Sleep quality was assessed with the Pittsburgh Sleep Quality Index (PSQI); a total score >7 indicated poor sleep. Physical activity was evaluated using the International Physical Activity Questionnaire Short Form (IPAQ-SF), and not meeting any moderate- or high-intensity criterion defined physical inactivity.

### 2.7 Statistical analysis

Statistical analyses were conducted using SPSS 27.0 (SPSS Inc., Chicago, IL) and R (version 4.5.2). Normally distributed continuous variables were expressed as mean (standard deviation), non-normally distributed data were shown as median (interquartile range), and categorical variables were reported as percentages. Differences among groups were assessed with *t*-tests, chi-square tests, and rank-sum tests. Logistic regression models were used to examine the association between BFP-defined obesity and depression. Three models were constructed. Model 1 was unadjusted. Model 2 was adjusted for age, sex, learning burden, drinking, and smoking. Model 3 was further adjusted for sleep quality and physical activity. The fully adjusted model was interpreted as estimating the independent association between BFP-defined obesity and depression conditional on these covariates. Statistical significance was set at a two-sided *P*-value < 0.05.

## 3 Results

### 3.1 Characteristics of the participants

Table 1 presents the baseline characteristics of the study participants. The study included 906 college students (354 males and 552 females). The mean age was 19.89 years, and the overall prevalence of G1, G2, and G3 was 9.71%, 8.28%, and 28.37%, respectively. The prevalence of depression was higher among obese students than among non-obese students (25.51% vs. 13.37%). Significant differences were observed among the four groups in age, sex, learning burden, sleep quality, smoking status, and physical activity, whereas drinking status did not differ significantly.

**Table 1:** Baseline characteristics of participants.

Variables	Overall	G1 (obese with depression)	G2 (non-obese with depression)	G3 (obese without depression)	G4 (non-obese without depression)	*P* value
*N* (cases)	906	88 (9.71)	75 (8.28)	257 (28.37)	486 (53.64)	
Age (years)	19.89 ± 1.69	19.26 ± 1.15	19.32 ± 1.43	19.44 ± 1.33	20.31 ± 1.84	**< 0.001**
Gender (%)						**< 0.001**
Male	354 (39.10)	6 (6.82)	27 (36.00)	41 (15.95)	280 (57.61)	
Female	552 (60.90)	82 (93.18)	48 (64.00)	216 (84.05)	206 (42.39)	
Learning burden (%)						**< 0.001**
Light	706 (77.93)	61 (69.32)	48 (64.00)	199 (77.43)	398 (81.89)	
Heavy	200 (22.07)	27 (30.68)	27 (36.00)	58 (22.57)	88 (18.11)	
Drinking (%)						0.140
Yes	314 (34.66)	36 (40.91)	33 (44.00)	87 (33.85)	158 (32.51)	
No	592 (65.34)	52 (59.09)	42 (56.00)	170 (66.15)	328 (67.49)	
Smoking (%)						**< 0.001**
Yes	207 (22.85)	12 (13.64)	15 (20.00)	23 (8.95)	157 (32.30)	
No	699 (77.15)	76 (86.36)	60 (80.00)	234 (91.05)	329 (67.70)	
Sleeping quality (%)						**< 0.001**
Good	583 (64.35)	35 (39.77)	16 (21.33)	169 (65.76)	363 (74.69)	
Poor	323 (35.65)	53 (60.23)	59 (78.67)	88 (34.24)	123 (25.31)	
Physical activity (%)						**< 0.001**
Sufficient	783 (86.42)	70 (79.55)	64 (85.33)	202 (78.60)	447 (91.98)	
Insufficient	123 (13.58)	18 (20.45)	11 (14.67)	55 (21.40)	39 (8.02)	

### 3.2 Association of obesity and depression

Logistic regression analyses showed that BFP-defined obesity was significantly association with depression in the overall population in the unadjusted model (OR = 2.22, 95% CI: 1.57–3.13, *P* < 0.05). After adjustment for age, sex, learning burden, drinking, and smoking, the association remained significant (OR = 1.58, 95% CI: 1.09–2.29, *P* < 0.05). Further adjustment for sleep quality and physical activity did not materially change the association (OR = 1.62, 95% CI: 1.10–2.38, *P* < 0.05). In sex-stratified analyses, this associated remained significant in females (Supplementary Table S1).

### 3.3 Gut microbiota description

Based on these findings, 104 participants were selected from the initial cohort for gut microbiota analysis and allocated into four groups: G1 (18.50 ± 0.72 years), G2 (18.93 ± 1.04 years), G3 (18.96 ± 1.26 years), and G4 (18.88 ± 0.95 years). As shown in Supplementary Table S2, no significant differences in age or sex distribution were found among the groups (all *P* > 0.05).

#### 3.3.1 . Alpha and beta diversity

Supplementary Table S3 displays the gut microbiota's richness and diversity across all groups. Several α-diversity indices, including Shannon, Simpson, Chao 1 and ACE revealed no significant difference in gut microbiota between group G1 and the three control groups (*P* > 0.05).

The PCoA analysis of β-diversity based on Bray-Curtis distance is displayed in Figure 2. Differences in microbial community structure were observed between G1 and G4 (*P* < 0.05), this indicates that group G1 exhibited a unique microbial composition compared to group G4 while the microbial composition of G1 resembled that of both the G2 and G3 groups (*P*_G1VS.G2_ = 0.063, *P*_G1VS.G3_ = 0.156).

**Figure 2:**
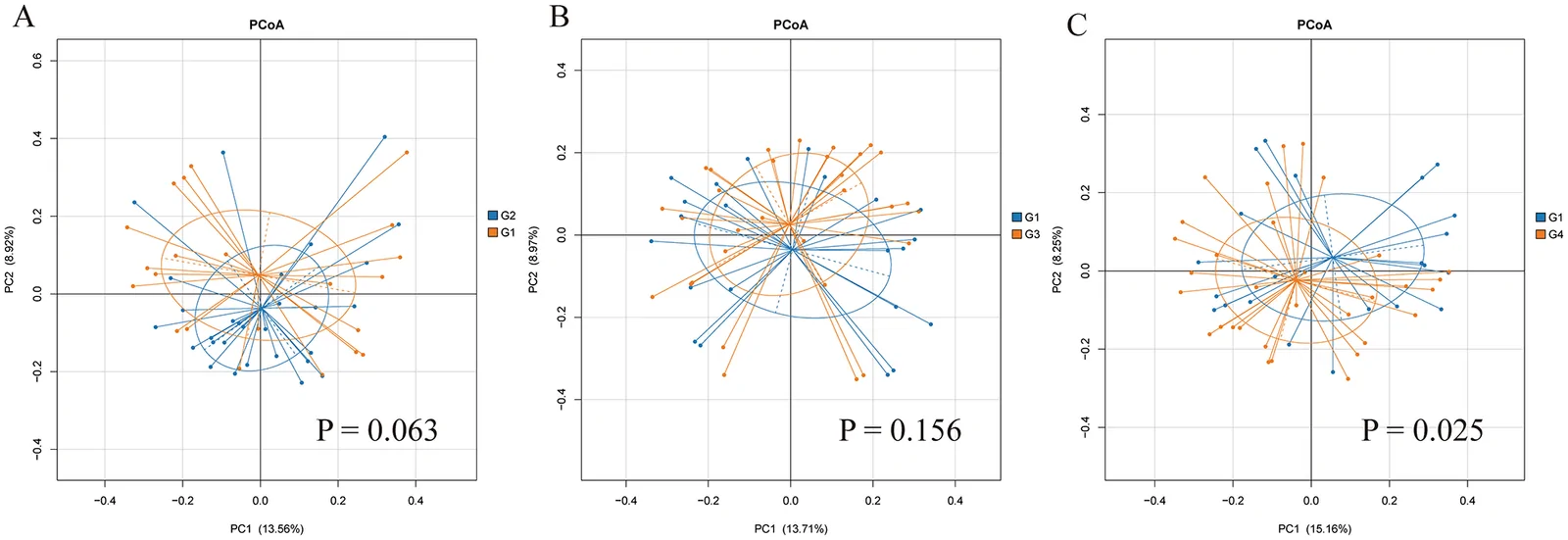
Principal coordinate analysis (PCoA) based on Bray-Curtis dissimilarity. **(A)**, G1: obese with depression vs. G2: non-obese with depression; **(B)**, G1: obese with depression vs. G3: obese without depression; **(C)**, G1: obese with depression vs. G4: non-obese without depression.

#### 3.3.2 Analysis of gut microbiota composition

Relative abundances of bacteria were compared at the phylum and genus taxonomic levels, and the bar chart of total species abundance showed species with relatively high overall abundance and their proportions in each group (Figure 3). At the phylum level, *Firmicutes, Actinobacteriota*, and *Bacteroidota* were the dominant phyla across all four groups. Compared with G2, G1 showed higher abundances of *Bacteroidota* and *Fusobacteriota*, and lower *Actinobacteriota*. Compared with G3 and G4, G1 had higher *Actinobacteriota* and *Fusobacteriota* but lower *firmicutes*.

**Figure 3:**
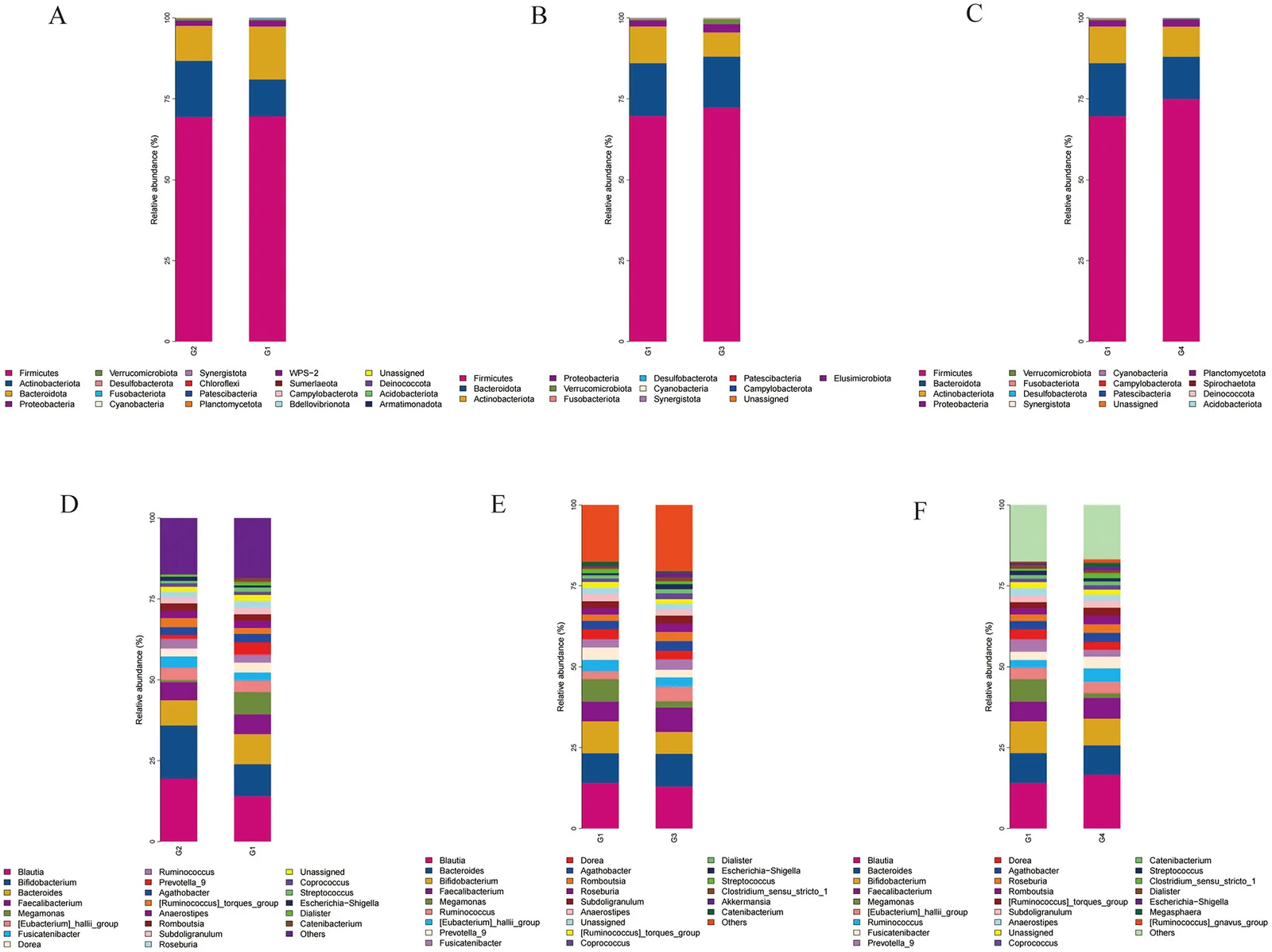
Relative abundance of bacterial taxa at the phylum and genus levels. **(A–C)** Phylum-level profiles and **(D–F)** genus-level profiles; **(A, D)** G1: obese with depression vs. G2: non-obese with depression; **(B, E)**, G1: obese with depression vs. G3: obese without depression; **(C, F)**, G1: obese with depression vs. G4: non-obese without depression. The bar plots are descriptive and do not indicate statistically significant differential abundance.

At the genus level, *Blautia, Bacteroides, Bifidobacterium, Faecalibacterium, Megamonas, Ruminococcus, Fusicatenibacter, and [Eubacterium]_hallii_group* were among the predominant genera across groups. Compared with G2, G1 showed lower relative abundance of *Blautia* and *Bifidobacterium*, but higher relative abundance of *Megamonas, Prevotella_9, Bacteroides, Collinsella*, and *Catenibacteriu*. Compared with G3, G1 showed higher relative abundance of *Bifidobacterium, Megamonas, Prevotella_9, Collinsella, Catenibacterium*, whereas *Akkermansia, Coprococcus, Faecalibacterium*, and *Ruminococcus* were lower. Compared with G4, G1 showed higher relative abundance of *Bifidobacterium, Megamonas, Prevotella_9, Collinsella*, and *Anaerostipes*, while *Blautia, Coprococcus, Faecalibacterium*, and *Ruminococcus* were lower.

#### 3.3.3 Candidate discriminative taxa among the analyzed groups

Linear discriminant analysis effect size (LEfSe) was used as an exploratory approach to identify taxa contributing most to the differences among groups, as shown in Figure 4. Compared with group G2, group G1 showed nominally higher relative abundance of 10 taxa, including *c_Negativicutes, o_Veillonellales_Selenomonadales*, and *g_Prevotella*, whereas 8 taxa, such as *s_Eubacterium_siraeum* and *g_Pyramidobacter* were identified as candidate taxa with higher relative abundance in group G2. Compared with group G3, 6 taxa, including *g_Stenotrophomonas* and *g_Burkholderia_Caballeronia_Paraburkholderia*, were identified as candidate taxa with higher relative abundance in group G1, while *g_Lachnospiraceae_NK4A136* showed a lower relative abundance trend in group G1. In the comparison between groups G1 and G4, 10 taxa were identified as candidate discriminative taxa in group G1, including *s_Bacteroides_faecis, s_Clostridium_spiroforme*, and *g_Lawsonella, o_Propionibacteriales, g_Butyricimonas, o_Veillonellales_Selenomonadales, c_Negativicutes*, whereas group G4 showed higher relative abundance trends of taxa such as *s_Bacteroides_nordii* and *g_Johnsonella*. However, after FDR correction, these taxa did not remain statistically significant; therefore, the LEfSe results should be interpreted as exploratory and hypothesis-generating.

**Figure 4:**
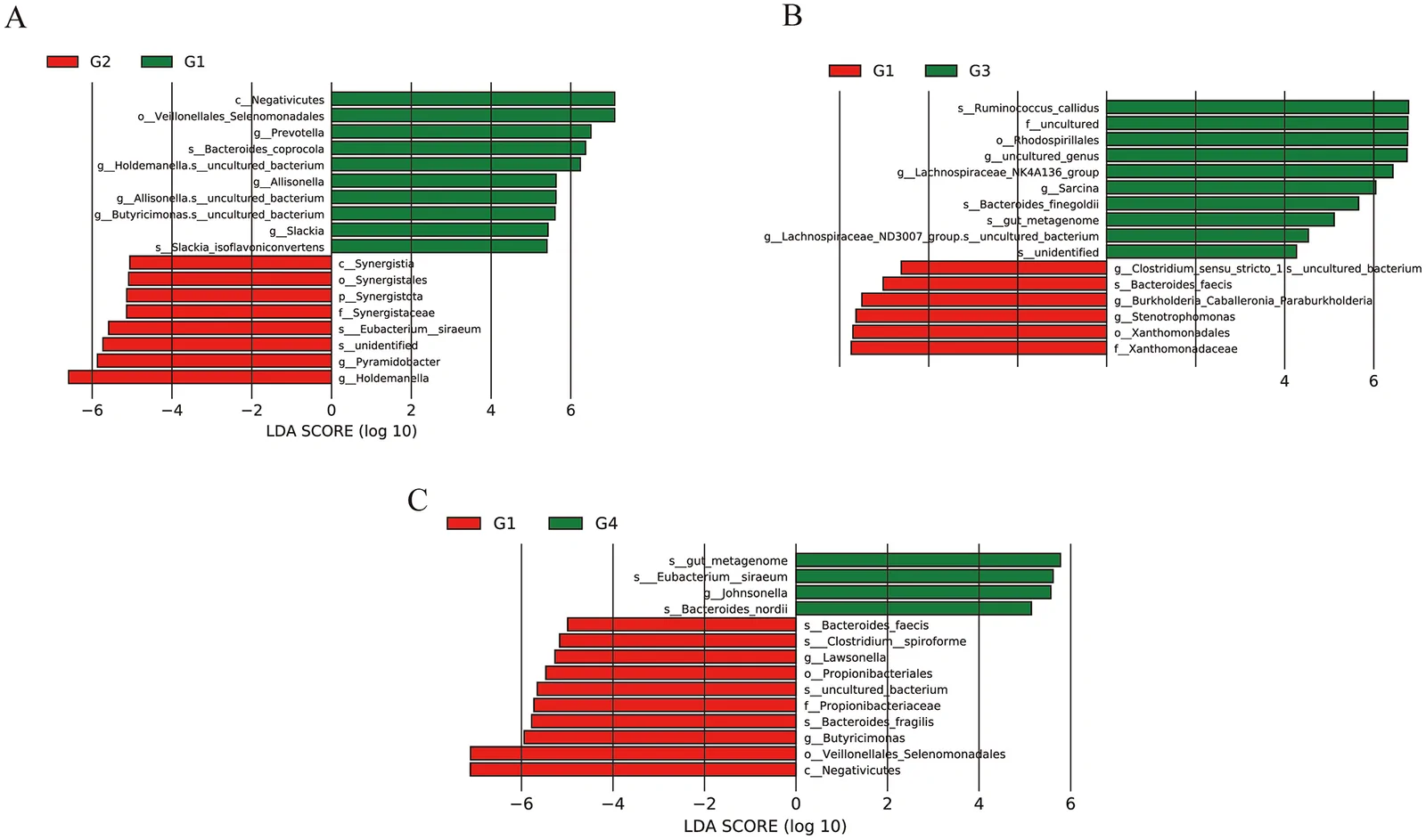
Candidate discriminative taxa identified by exploratory LEfSe analyses. Taxa with nominal *P* < 0.05 and an LDA score (log10) >2 are shown. These taxa did not remain significant after FDR correction and should therefore be interpreted as exploratory candidate features. **(A)**, G1: obese with depression vs. G2: non-obese with depression; **(B)**, G1: obese with depression vs. G3: obese without depression; **(C)**, G1: obese with depression vs. G4: non-obese without depression.

#### 3.3.4 Functional prediction analysis

PICRUSt-based KEGG pathway prediction suggested nominal differences in inferred microbial functional potential between G1 and the control groups (Figure 5). These results should be interpreted as exploratory predictions based on 16S rRNA profiles rather than directly measured functional activity. Compared with G2, five predicted KEGG pathways showed higher inferred abundance in G1: including lipopolysaccharide biosynthesis (ko00540), D-arginine and D-ornithine metabolism (ko00472), geraniol degradation (ko00281), and linoleic acid metabolism (ko00591) were upregulated. In G1 vs. G3, two predicted pathways showed higher inferred abundance in G1, including tetracycline biosynthesis (ko00253) and bisphenol degradation (ko00363). When comparing G1 with G4, several predicted pathways showed higher inferred abundance in G1, including the phosphotransferase system (PTS, ko02060), lipopolysaccharide biosynthesis (ko00540), D-arginine and D-ornithine metabolism (ko00472), lipoic acid metabolism (ko00785), and valine, leucine and isoleucine degradation (ko00280).

**Figure 5:**
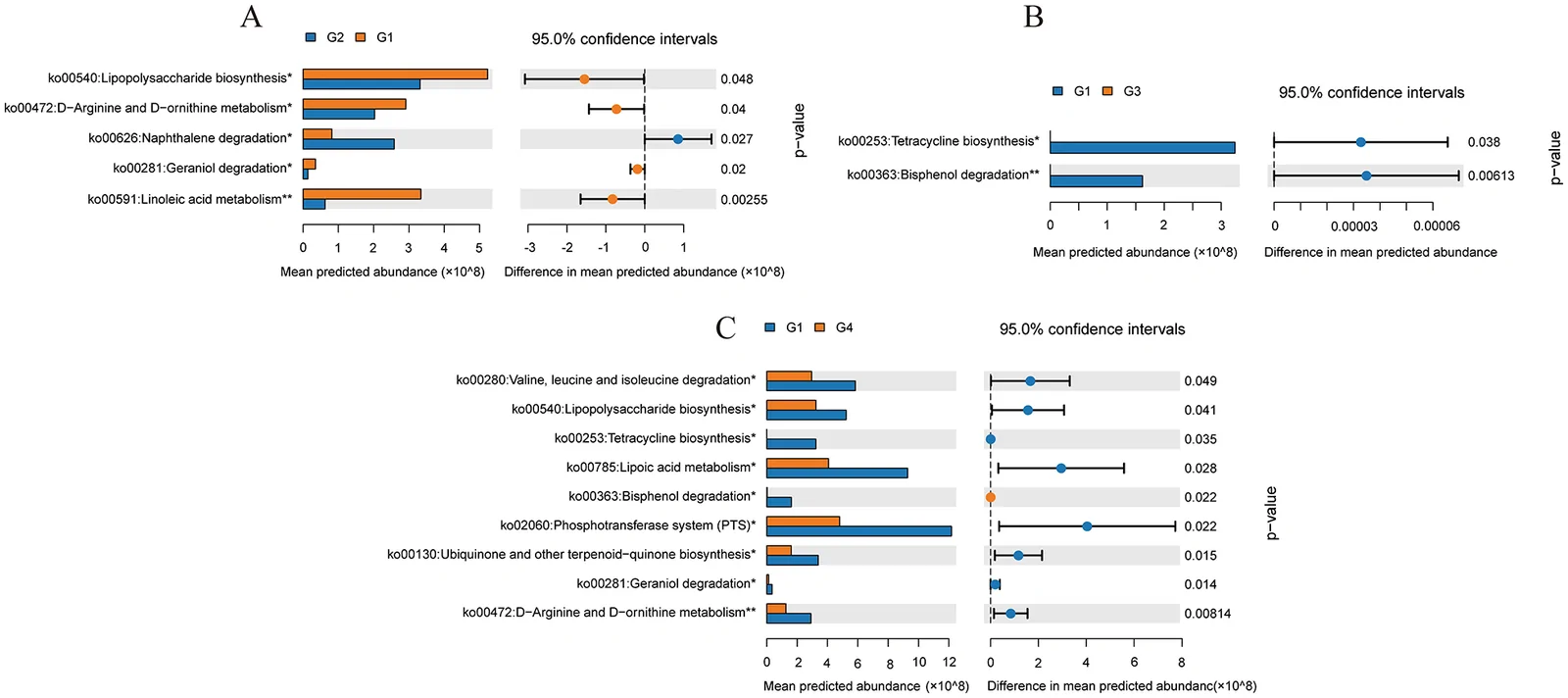
Exploratory PICRUSt-predicted KEGG pathways in G1 and the comparison groups. Extended error-bar plots show pathways with nominal *P* < 0.05 in pairwise comparisons. These results represent predicted microbial functional potential derived from 16S rRNA profiles and should not be interpreted as FDR-controlled findings or direct functional measurements. **(A)**, G1: obese with depression vs. G2: non-obese with depression; **(B)**, G1: obese with depression vs. G3: obese without depression; **(C)**, G1: obese with depression vs. G4: non-obese without depression.

## 4 Discussion

This cross-sectional study examined the co-occurrence of obesity and depression among college students and explored associated gut microbiota features. Co-occurring obesity and depression were present in 9.71% of participants, and BFP-defined obesity was associated with higher odds of depression after covariate adjustment. In the microbiome subset, alpha diversity did not differ among groups, whereas beta diversity differed between G1 and G4. At the genus level, the comorbid group showed higher relative abundance *Fusobacterium* and *Collinsella*, and lower relative abundance *Akkermansia* and *Coprococcus*. Functional prediction also suggested a higher inferred abundance of the lipopolysaccharide biosynthesis pathway in the comorbid group. Overall, these findings are consistent with the possibility of an inflammation-related microbial pattern in the comorbid group, although this interpretation remains exploratory.

The association between BFP-defined obesity and depression is an important finding of this study. Previous studies have reported positive associations between BFP and depressive symptoms in different populations. For example, Li et al. found that total BFP was significantly associated with depression defined by the PHQ-9 in the general U.S. population (24). Similarly, Gu et al. reported that individuals in the highest quartile of body fat percentage had an increased likelihood of depression (25). However, evidence focusing specifically on college students remains limited. In the present study, obese students had higher odds of depression than non-obese students after adjustment for potential confounders. This finding may be related to the unique psychosocial and lifestyle characteristics of college students, including academic stress, irregular sleep, insufficient physical activity, and unhealthy dietary behaviors (26, 27). In sex-stratified analyses, the association remained significant in females but not in males, which is consistent with some previous studies suggesting that adiposity-related psychological vulnerability may be more pronounced in females (28).

Another finding of this study was that students with comorbid obesity and depression showed differences in gut microbiota composition compared with other groups, although these microbiome results should be considered exploratory. While α-diversity indices were not significantly different among groups, β-diversity differed significantly between the comorbid group and healthy controls, indicating that the overall microbial community structure may be altered in the comorbid state. Previous studies have reported reduced microbial diversity in individuals with obesity or depression (14, 29); however, findings remain inconsistent across populations. The absence of significant differences in α-diversity in our study may be related to the relatively young and homogeneous study population, limited sample size, and approximate balance in age and sex. At the phylum level, the bar plots suggested that G1 had a relatively higher abundance of *Fusobacteriota* across all three pairwise comparisons. At the genus level, G1 appeared to have higher relative abundances of *Megamonas, Collinsella*, and *Prevotella_9*, together with a lower relative abundance of *Coprococcus*, across all comparisons. The consistently higher *Collinsella* in G1 may be noteworthy because previous studies have linked *Collinsella* to inflammatory activity and altered gut permeability (30). *Fusobacterium* was also descriptively more abundant in G1 than in G3, and experimental studies have linked *Fusobacterium nucleatum* to pro-inflammatory signaling and intestinal inflammation (31, 32). By contrast, *Akkermansia* was lower in G1 than in G3, and *Coprococcus* was lower in G1 than in both G3 and G4. Prior literature has discussed *Akkermansia muciniphila* in relation to intestinal barrier integrity and immune regulation (33) and *Coprococcus* as a butyrate-producing genus potentially relevant to depression-associated microbiome profiles (34). These prior findings provide biological context but do not demonstrate inflammatory activity or causal mechanisms in our participants. Moreover, *Prevotella*-related profiles are associated with long-term dietary patterns (35). This point is important in our study because only age and sex were approximately balanced across the microbiome subgroups, whereas diet and other potential confounders were not assessed or adjusted for. Diet may simultaneously affect the gut microbiota, obesity and depression (36–38). Thus, the bar plots illustrate descriptive compositional differences but do not establish absolute microbial expansion, depletion, specificity to depression or obesity, or causality.

LEfSe analysis was further performed to explore candidate taxa that may contribute to microbial differences in college students with obesity and depression. In the G1 group, several taxa, including *s_Bacteroides_faecis, s_Clostridium_spiroforme, g_Butyricimonas, o_Veillonellales_Selenomonadales* and *c_Negativicutes* were identified as candidate discriminative features compared with G4. Some of these taxa have previously been associated with intestinal inflammation or metabolic disturbances. For example, *Clostridium spiroforme* and *Lawsonella* have been linked to intestinal inflammation or metabolic disturbances in previous studies, although their roles in obesity-depression comorbidity remain unclear (39, 40). However, it should be noted that these LEfSe-identified taxa did not remain significant after FDR correction. Therefore, these findings should be interpreted cautiously as exploratory observations rather than confirmed microbial biomarkers. PICRUSt predicted a higher abundance of the lipopolysaccharide (LPS) biosynthesis pathway in G1 than in G2 and G4. This finding represents inferred functional potential from 16S rRNA profiles, not evidence of increased LPS production or inflammatory activation. In previous studies, LPS, a major component of the outer membrane of Gram-negative bacteria, has been shown to activate Toll-like receptor 4 and promote the release of pro-inflammatory cytokines, including IL-1β, IL-6, and TNF-α(41). Previous studies have also suggested that LPS may activate IDO, thereby altering tryptophan metabolism, reducing 5-HT availability in the central nervous system, and potentially contributing to depressive symptoms (9). These literature-based mechanisms provide context for hypothesis generation only. Circulating LPS, inflammatory biomarkers, metagenomic functions, and metabolomic profiles were not measured, and causal inference is not possible in this cross-sectional study.

Several limitations should be considered. First, the cross-sectional design precludes causal inference. Second, the microbiome subset was small, limiting statistical power; consequently, the microbiome findings are exploratory and require validation in larger independent cohorts. Third, the microbiome groups were selected to achieve approximate balance only in age and sex. Dietary intake and other potential confounders were not assessed or controlled, and differences in fiber, fat, or other dietary exposures may have contributed to the observed microbiota patterns. Fourth, depression was defined by PHQ-9 ≥10 alone, without additional instruments for comprehensive evaluation; although validated, the PHQ-9 cannot replace clinical diagnosis. Fifth, inflammatory biomarkers were not measured, and microbial functions were predicted from 16S rRNA data rather than directly assayed; thus, these findings are hypothesis-generating and warrant future studies using metagenomics, metabolomics, and inflammatory biomarkers. Finally, participants were recruited from northwest China, limiting generalizability to other populations or regions.

In conclusion, this study found that comorbid obesity and depression were present in a considerable proportion of college students and that body fat percentage -defined obesity was associated with depression. Co-occurring obesity and depression were also associated with exploratory differences in gut microbial composition and predicted functional potential; however, no taxa remained significant in the FDR-controlled ANCOM-BC2 analyses, and the functional findings were based on PICRUSt predictions. These observations do not establish causality or inflammatory activation. Larger longitudinal studies incorporating detailed dietary assessment, direct inflammatory measurements, metagenomics, and metabolomics are needed to clarify these relationships.

## Data Availability

The datasets presented in this study can be found in online repositories. The name of the repository and accession number can be found here: National Center for Biotechnology Information (NCBI) BioProject, https://www.ncbi.nlm.nih.gov/bioproject/, PRJNA1503426.
