# Supplementary material for: Associations of body fat percentage-based obesity with depression and comorbidity-related gut microbiota features in college students
Source: Front Nutr. 2026 Sep 17;13:1906822. doi: 10.3389/fnut.2026.1906822 (PMC13627446; doi:10.3389/fnut.2026.1906822)
Supplement: Supplementary file 1 [file Supplementary_file_1.pdf]

Supplementary Material

1 Supplementary Tables

Table S1 Association between BFP-defined obesity and depression

	Model 1		Model 2		Model 3	
	OR (95% CI)	*P* value	OR (95% CI)	*P* value	OR (95% CI)	*P* value
Body fat percentage						
Non-obese	Ref		Ref		Ref	
Obese	2.22(1.57, 3.13)	**<0.001**	1.58(1.09, 2.29)	**0.016**	1.62(1.10, 2.38)	**0.015**
Male body fat percentage						
Non-obese	Ref		Ref		Ref	
Obese	1.52(0.59, 3.90)	0.386	0.70(0.54, 1.91)	0.481	0.59(0.19, 1.78)	0.345
Female body fat percentage						
Non-obese	Ref		Ref		Ref	
Obese	1.63(1.09, 2.44)	**0.018**	1.65(1.09, 2.49)	**0.018**	1.74(1.13, 2.67)	**0.012**

BFP, body fat percentage; Model 1, unadjusted model; Model 2, adjusted for age, gender, learning burden, drinking, smoking; Model 3, was further adjusted for sleep quality and physical activity.

Table S2 Demographic features of obese individuals with depression and control groups

Variables	G1 (obese with depression)	G2 (non-obese with depression)	G3 (obese without depression)	G4 (non-obese without depression)	*P* value
Age	18.50±0.72	18.93±1.04	18.96±1.26	18.88±0.95	0.353
Gender					0.907
Male	6	8	8	9	

Female	18	19	19	17

Table S3 Exploratory alpha-diversity comparisons between G1 and comparison groups

Index	*P* (G1vs G2)	*P* (G1 vs G3)	*P* (G1 vs G4)
Observed	0.270	0.139	0.965
Chao1	0.274	0.142	0.979
ACE	0.268	0.152	0.993
Shannon	0.201	0.549	0.855
Simpson	0.208	0.468	0.896
Coverage	0.041	0.871	0.413
